# Comparative analysis of dose-response variability and severity in STZ-induced diabetes: female vs. male NSG mice

**DOI:** 10.1038/s41598-026-42408-z

**Published:** 2026-03-05

**Authors:** Steven R. Talbot, Miriam Heider, Martin Wirth, Anne Jörns, Ortwin Naujok

**Affiliations:** 1 Hannover Medical School, Institute of Clinical Biochemistry, Hannover, Germany; 2 Hannover Medical School, Institute for Laboratory Animal Science, Hannover, Germany

**Keywords:** Female NSG mice, Diabetes, Streptozotocin, Severity assessment, RELSA, Diseases, Endocrinology, Physiology

## Abstract

This study evaluated the dose–response relationship of streptozotocin in female NSG mice to identify a dose that reliably induces diabetes mellitus while minimizing animal burden. Although streptozotocin is widely used to model insulin-dependent diabetes in rodents, sex-specific responses are underexplored, and female mice are often excluded due to their reduced sensitivity. We tested single doses ranging from 125 to 225 mg/kg body weight. Concentrations of 125 and 150 mg/kg yielded insufficient diabetes induction, whereas 200 and 225 mg/kg caused rapid, severe hyperglycemia and dramatic weight loss requiring early termination. A dose of 175 mg/kg body weight streptozotocin emerged as optimal, inducing stable hyperglycemia in approximately 90% of the animals with acceptable weight loss and minimal mortality. Compared to males, females required ~ 25 mg more streptozotocin for comparable outcomes, reflecting lower vulnerability likely linked to estrogen signaling. We did not observe significant differences in terms of animal suffering when comparing female to male mice. Additionally, sex-specific diagnostic thresholds for hyperglycemia were identified. These results provide the first dose-response data for female NSG mice, offering refined guidance for preclinical diabetes research and supporting the inclusion of both sexes in experimental design.

**Supplementary Information:**

The online version contains supplementary material available at 10.1038/s41598-026-42408-z.

## Introduction

Biomedical research has historically relied on animal models. In experimental diabetes mellitus (DM) research, NOD.Cg-Prkdc^scid^ Il2rg^tm1Wjl^/SzJ (NSG) mice are frequently used as a preclinical animal model for xenotransplantation experiments using human islets or islets from other species^1,2^. To induce diabetes, streptozotocin (STZ), a highly selective beta cell toxin, is administered beforehand either through a single-dose or multiple low-dose injection regimen^3^. However, female mice are less prone to STZ-induced beta cell toxicity, display more variable metabolic outcomes and infrequent partial recovery of glucose tolerance when compared to male mice^4–7^. According to the data available so far, this might be caused by to the sex hormone estrogen, which is particularly high in the proestrus cycle and has been shown to protect beta cells from STZ toxicity^8^. For reasons of feasibility this has historically led to more experimental studies using male than female mice. However, with regard to the approval of clinical trials in humans, national regulatory authorities require sex to be considered as a biological variable.

In type 1 and type 2 DM sex-specific differences are known both during therapy and the development of chronic illnesses. Female patients are reported to be affected by changes in insulin sensitivity during the menstrual cycle^9–11^, pregnancy^12,13^, and menopause^14^. Furthermore, glucose-lowering drugs are more effective in women than in men^15^. With respect to late-stage organ failures, females compared to males show an increased cardiovascular risk profile^16^, a higher incidence of stroke^17^, and a higher incidence of coronary heart disease^18^ and heart failure^19^. This adds up to a loss of 17.7 life-years for women; 3.5 years more than men if type 1 DM manifested early in life^20^.

We have recently explored the dose-response variability and relative severity of STZ treatment in male NSG mice to define an optimal single dose of STZ that not only reliably induces diabetes but also limits weight loss and animal suffering^21^. In the present study, with the same objectives, female NSG mice were examined in an STZ dose-response curve ranging from 125 to 225 mg/kg body weight. To minimize estrogen-induced resistance against STZ^8^, only mice outside the proestrus cycle were used.

The RELSA algorithm^22^, a multidimensional, quantitative calculation method for assessing the severity of animal burden in experimental procedures, was employed to calculate animal suffering. Finally, data from this report using female mice were compared with the data from male mice from our previous study^21^ to compare the efficacy and stress of STZ treatment between males and females. Machine learning was employed to calculate optimal thresholds for distinguishing between healthy and diabetic animals of both sexes, as well as to determine the optimal thresholds for diabetes classification. It turned out that female mice were apparently more resilient than male mice towards STZ-induced beta cell destruction, resulting in a slightly shifted dose-response curve compared to our previous data. Similarly, we can demonstrate that female NSG mice, despite their greater resilience to STZ, are a suitable and reliable model for studying diabetes.

## Results

### Dose-dependent effect of STZ on blood glucose levels in female NSG mice

**Fig. 1:**
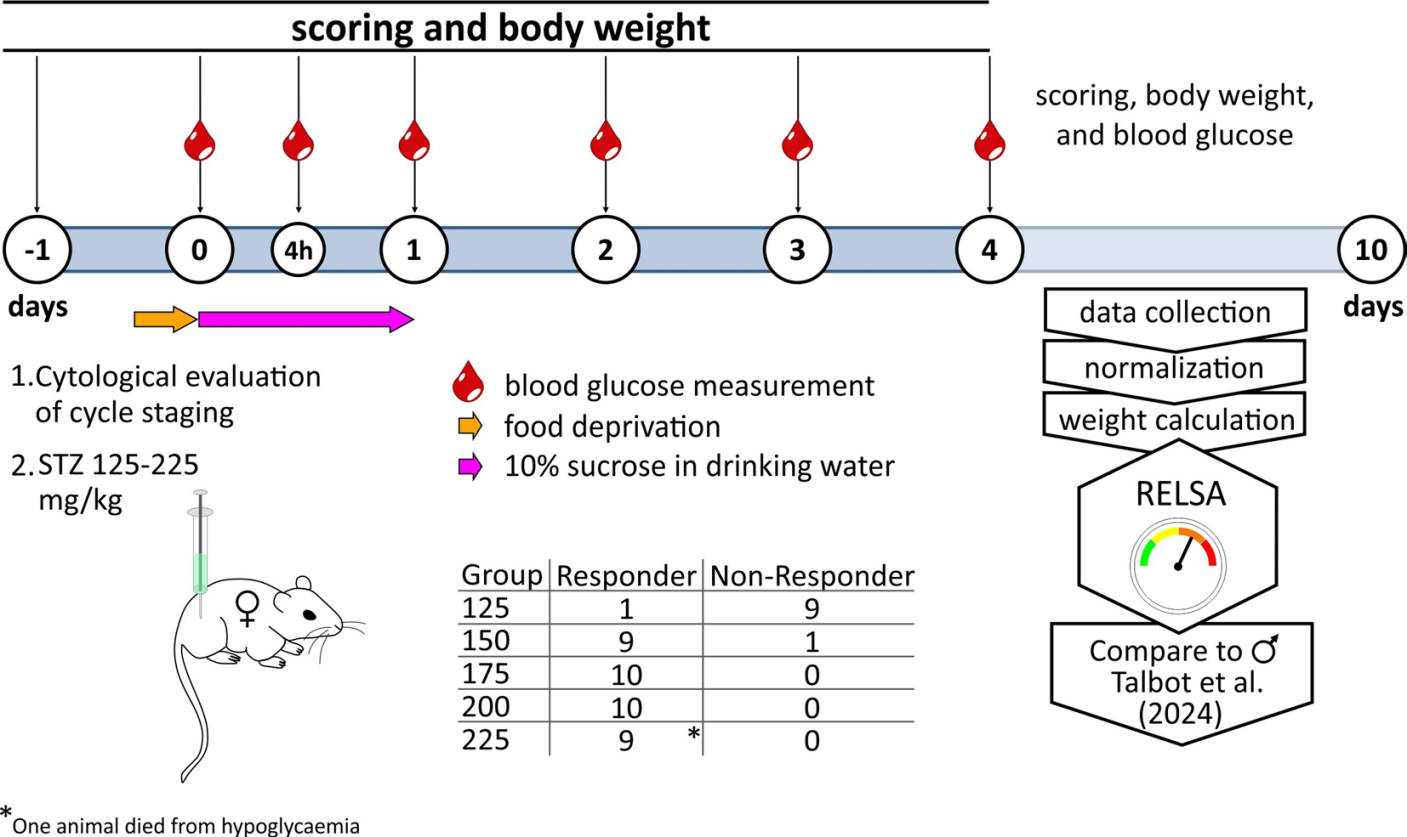
Schematic presentation of the experimental proceedings.

Streptozotocin is a potent toxin causing pancreatic beta cell destruction after it has been taken up by the cells via the GLUT2 glucose transporter from the bloodstream. The resulting absolute insulin deficiency leads to hyperglycemia and weight loss. Hence, these two parameters were addressed in this study. Estrogen-related resistance against STZ is a mechanism by which females are partially protected against this diabetogenic compound^8^. Therefore, before STZ injection, a vaginal cytological examination was performed to determine the cycle status. Female NOD.Cg-Prkdc^scid^ Il2rg^tm1Wjl^/SzJ (NSG) mice found to be in the pro-oestrus cycle with peak serum estrogen were spared the injection and postponed to a later injection date (Fig. 1).

Baseline values of the mice were a body weight of 23 ± 1.3 g (SD, *n* = 60), 5.4 ± 1.0 mmol/L (SD, *n* = 60) fasting blood glucose (FBG; after 4 h fasting), and 6.6 ± 0.8 mmol/L random blood glucose (RBG) in 10 control animals, 82 measurements. Supplementary Fig. 1 compares the baseline values to those of male mice from our previous study^21^.

**Fig. 2:**
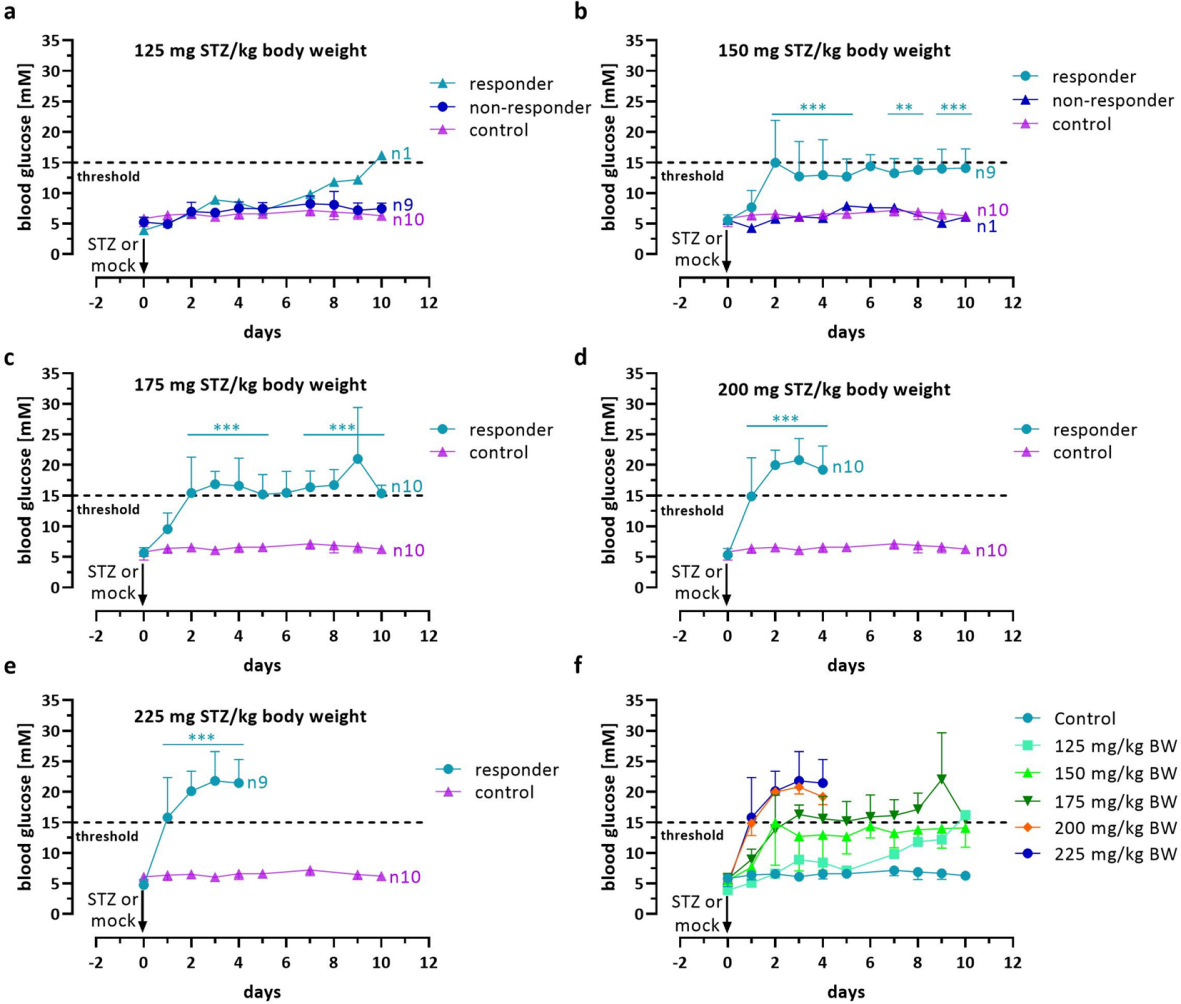
Dose-dependent effect of STZ on blood glucose in female NSG mice. Changes in blood glucose values in mmol/L over 10 days in STZ-treated female NSG mice are shown vs. mock controls. The mice were injected i.p. with 125 (**a**), 150 (**b**), 175 (**c**), 200 (**d**), or 225 mg/kg STZ per kg body weight (**e**). Separate curves are presented for diabetic (responders) and non-diabetic animals (non-responders). Data are presented as means ± SD, *n* = 10. The groups were compared on individual days with *Student’s t*-test, corrected for multiple comparisons by the Bonferroni-Dunn method, resulting in adjusted p-values: *** *p* ≤ 0.001, ** *p* ≤ 0.01, * *p* ≤ 0.05. In (**f**), a summary of the blood glucose changes of all diabetic animals (responders) is presented. The dotted line indicates the threshold for diabetes induction after two consecutive blood glucose measurements above 15 mmol/L.

In female NSG mice, a single-dose i.p. injection of different STZ concentrations, ranging from 125 to 225 mg/kg STZ per kg body weight (abbreviated as mg/kg STZ) was performed, resulting in a reliable chemical induction of diabetes in three out of five concentrations (Fig. 2).

Injection of 125 mg/kg STZ caused, in most mice, a slight non-significant increase in blood glucose compared to the controls (Fig. 2a). Only one mouse in this group showed a deteriorating blood glucose curve and was measured on the last day of the experiment with a blood glucose concentration above 15 mmol/L. In contrast, the STZ concentration of 150 mg/kg resulted in increased blood glucose levels in 9 out of 10 animals. However, these concentrations did not consistently exceed the threshold of 15 mmol/L, which we had previously set as a diagnostic criterion for diabetes. Instead, the blood glucose concentrations in this group fluctuated between 12.3 and 14.7 mmol/L. Still, they were significantly higher (*** *p* < 0.0008, ANOVA plus *Dunnett’s* post hoc test) from day 2 of the experiment compared to the FBG on day 0 (Fig. 2b). When using 175 mg/kg STZ, all mice, still one, became diabetic 48 h after injection, with values slightly higher than those observed with 150 mg/kg STZ, but again, the threshold of 15 mmol/L was not consistently reached. One animal exhibited highly variable blood glucose concentrations, ranging from 8.2 to 18.7 mmol/L, but primarily remained below the 15 mmol/L threshold in 9 out of 10 measurements (11.5 ± 3.6 mmol/L, 10 data points) (Fig. 2c).

The two highest STZ concentrations of 200 and 225 mg/kg STZ, however, led to a fulminant and complete manifestation of diabetes in all animals, typically already after 24 h but at the latest two days after injection (Fig. 2d/e). One animal in the 225 mg/kg STZ group died of a hypoglycaemia-related event within 24 h, though. Figure 2f presents a summary of blood glucose curves for all responding animals, illustrating the correlation between the STZ concentration and the dynamics of diabetes development.

**Fig. 3:**
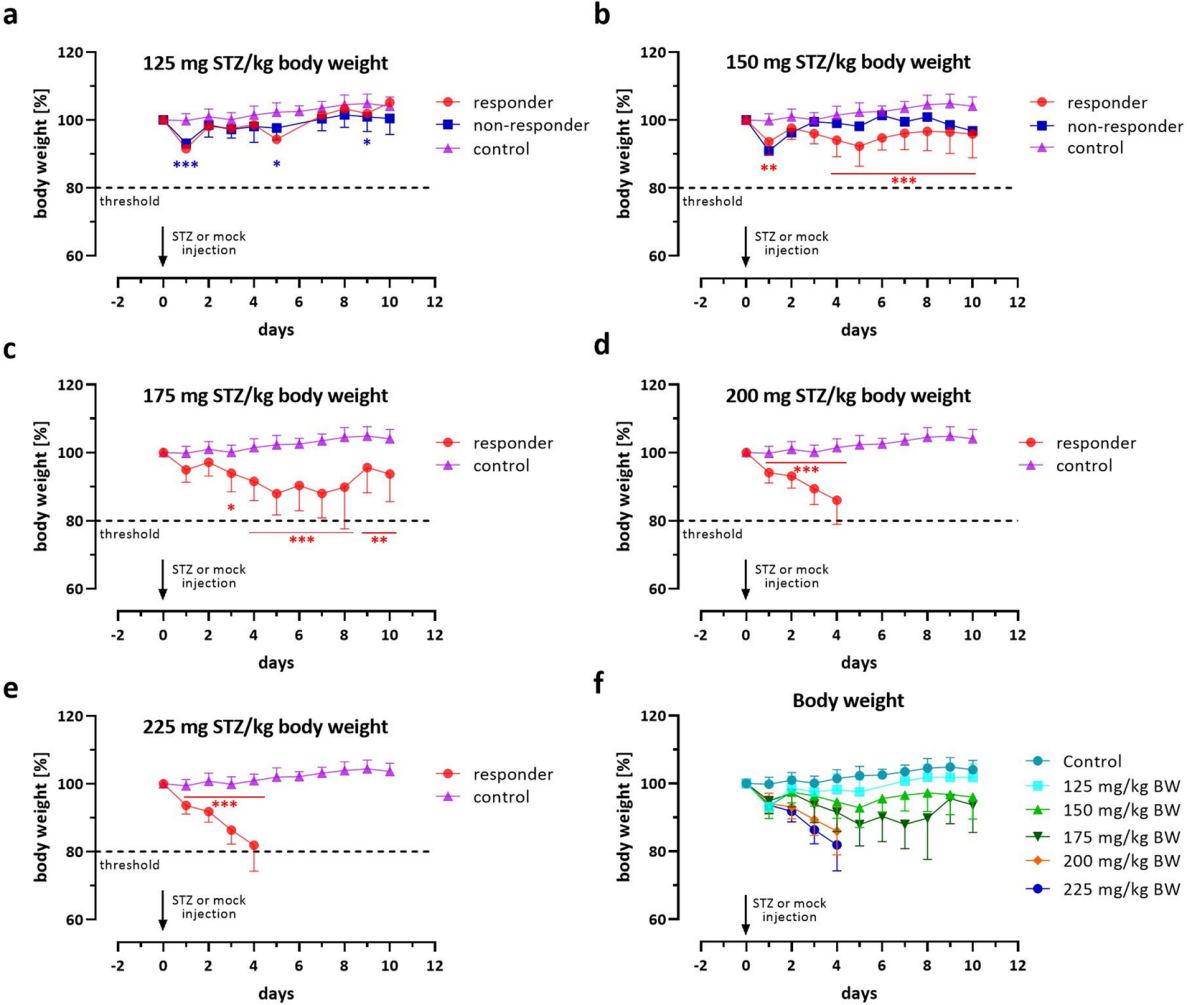
Dose-dependent effect of STZ on the body weight of female NSG mice. Shown are changes in body weight in percentage to the baseline weight (calculated from day − 1 and day 0) over 10 days of STZ-treated NSG mice vs. mock controls. The mice were injected i.p. with 125 (**a**), 150 (**b**), 175 (**c**), 200 (**d**), and 225 mg/kg STZ per kg body weight (**e**). Separate curves are presented for diabetic (responders) and non-diabetic animals (non-responders). Data are presented as means ± SD, *n* = 9–10. The groups were compared on individual days with *Student’s t*-test, corrected for multiple comparisons by the Bonferroni-Dunn method, resulting in adjusted p-values: *** *p* ≤ 0.001, ** *p* ≤ 0.01, * *p* ≤ 0.05. In (**f**), a summary of the changes in body weights for all diabetic animals (responders) is presented. The dotted line marks the 20% weight loss threshold value that indicates a body condition score that requires euthanasia.

All mice showed an immediate weight loss of 6.4% ± 0.9 (SD, *n* = 49) percent within 24 h of the injection, except for the mock-injected controls that retained their weight (Fig. 3a-e). In the group of 125 mg/kg STZ, the body weight curves for diabetic (responders) and non-diabetic animals (controls and non-responders) were very similar overall. They showed a quick recovery from the initial weight loss (Fig. 3a).

Injection of 150 mg/kg STZ resulted in a slight weight loss over the course of the experiment, which became significant on day 4 (Fig. 3b). At 175 mg/kg, a considerable weight loss of more than 10% was observed within the first 6–8 days. From that point, some diabetic mice showed either a stabilization or a recovery of their body weight despite their diabetic condition. Still, at the end of the experiment, the body weight was significantly reduced compared to the control (Fig. 3c).

**Fig. 4:**
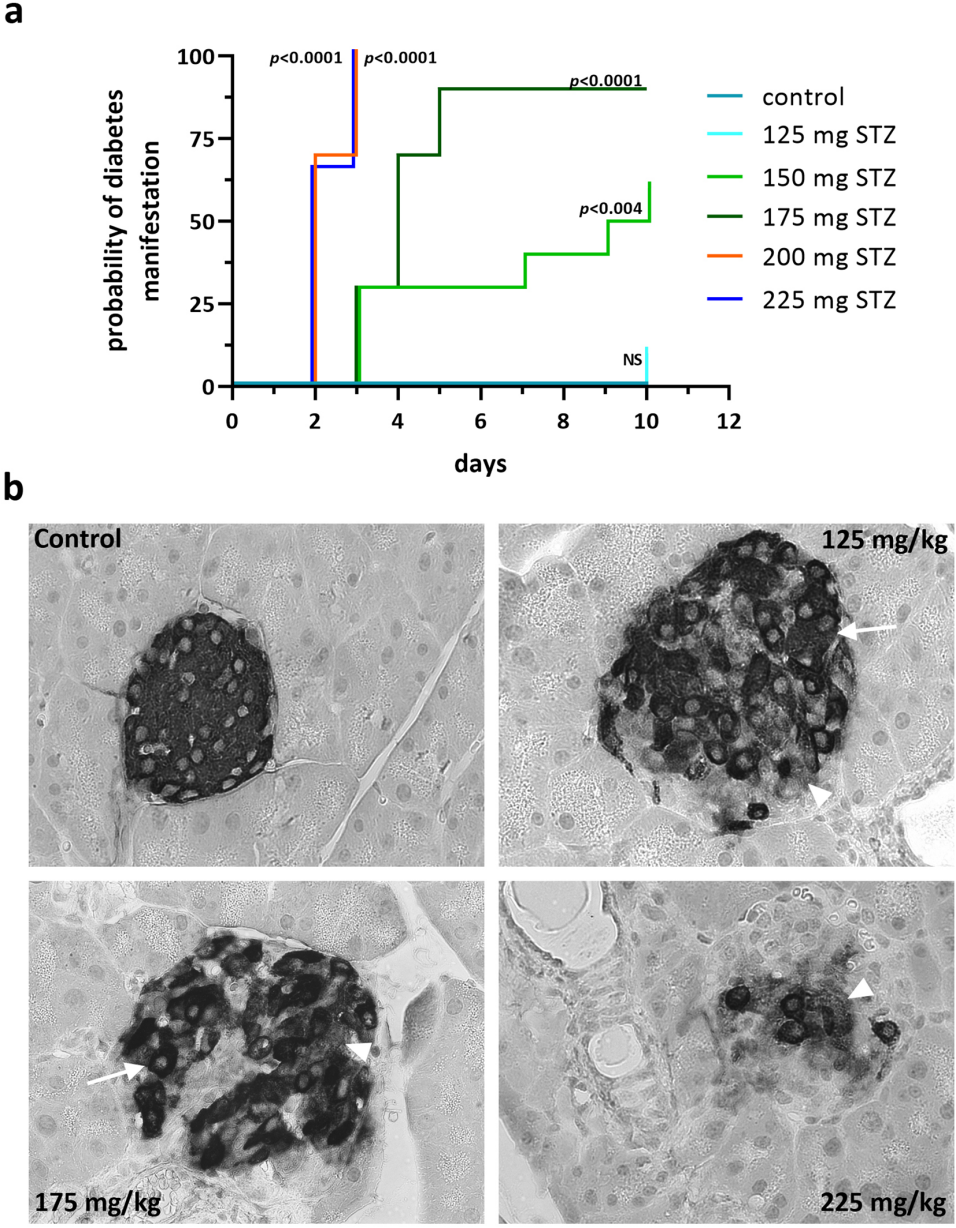
Kaplan-Meier survival analysis and changes of beta cell survival after STZ treatment. (**a**) The probability of diabetes manifestation (15 mmol/L threshold) in female NSG mice after treatment with STZ ranged from 125–225 mg/kg STZ per kg body weight compared to the mock control (in teal) over the time course of the study. Statistical differences were calculated using the Log-rank (Mantel-Cox) test. (**b**) Shown is an immunohistochemical staining of insulin of pancreatic sections obtained from vehicle-treated NSG mice, NSG mice with 125 mg STZ/kg body weight, 175 mg STZ/kg body weight, 225 mg STZ/kg body weight. Arrows point on densely stained beta cells, arrowheads denote to degranulated beta cells with faint insulin staining. 40x magnification.

The two high STZ concentrations, however, led to a fulminant and continuous loss of body weight in all animals, predominantly at 225 mg/kg STZ (86.0% ± 7.0 and 81.9% ± 7.6 [SD], *n* = 9–10, mean body weight compared to the baseline) within 96 h after STZ injection (Fig. 3d/e). This required premature termination of the experiment in many cases, as the body weight fell below the 80% threshold line. Figure 3f presents a summary of all body weight curves for all responding animals.

The survival analysis summarizes the response of female NSG mice to different STZ concentrations over time (Fig. 4a). A threshold value of 15 mmol/L was set as the criterion for successful diabetes manifestation to ensure comparability with our previous study^21^. A rapid induction of diabetes was observed in the 200 and 225 mg/kg STZ groups. In comparison, at 175 mg/kg and 150 mg/kg STZ, high blood glucose levels occurred with a significant delay, whereas 125 mg/kg STZ proved unreliable (Fig. 4a).

In control animals without STZ treatment the islets were well preserved and all beta cells were densely immunostained for insulin (Fig. 4b). The treatment with 125 mg STZ caused a moderate reduction of densely stained beta cells. In addition, a substantial number of beta cells was degranulated. Beta cell loss after treatment with 175 mg/kg STZ further decreased the number of densely stained cells. A significant portion of the islets showed diffuse and faint insulin staining. Only a few insulin-positive beta cells with a dense and a little more beta cells with a faint insulin staining remained at the highest concentration of 225 mg/kg STZ (Fig. 4b).

### Dose and time-dependent characteristics of female NSG mice

**Fig. 5:**
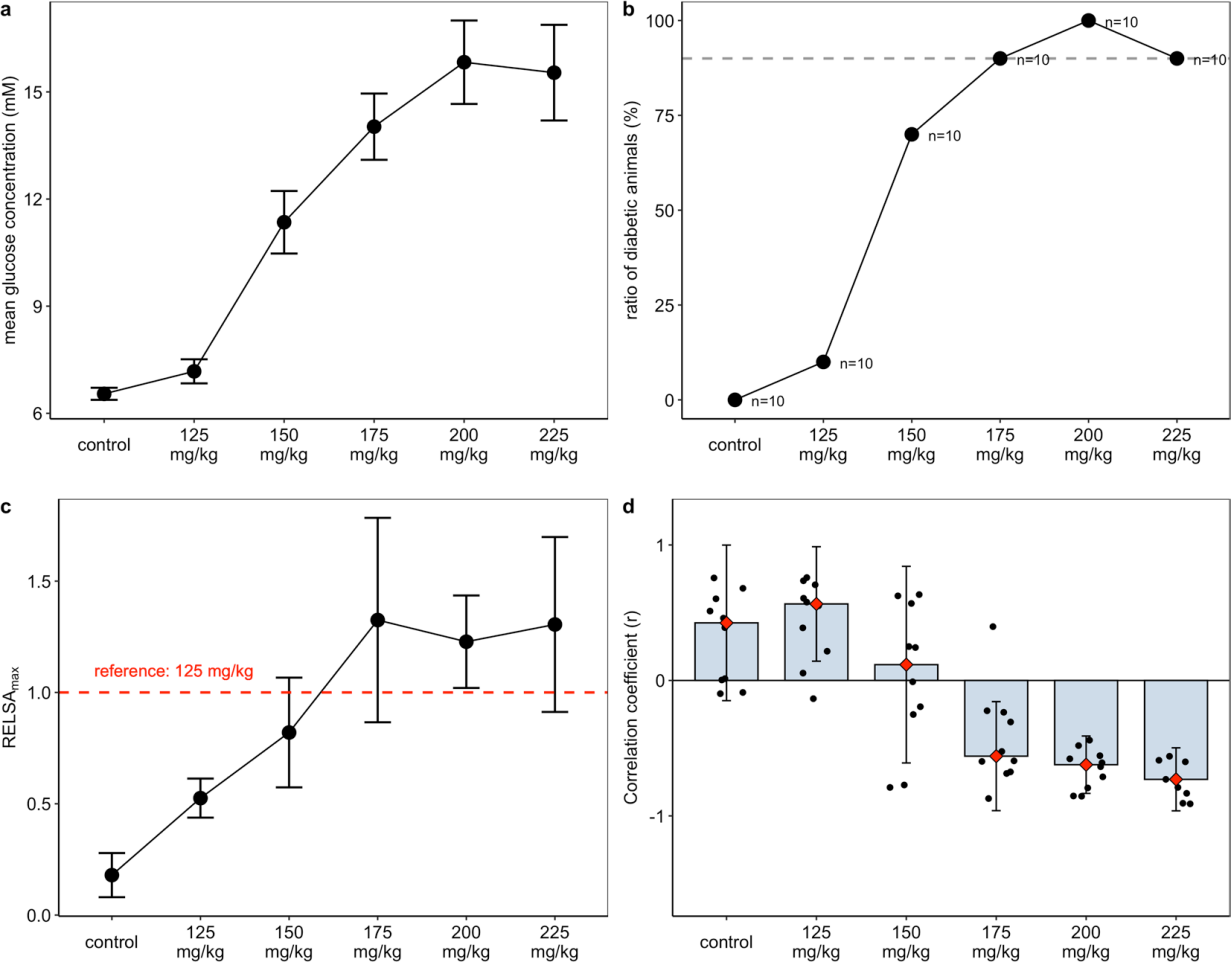
(**a**) shows the average time-independent blood glucose concentration per STZ dose in female NSG mice. The roughly sigmoidal curve indicated that for each unit increase in glucose (mM), the Odds Ratio in favor of the diabetic event was 1.56 (CI_95%_ [1.45, 1.72]), which was larger than that in male mice (OR = 1.44) reported in our previous study^21^. This means that the strength of the association between glucose and diabetes is slightly stronger in females than in males. Analyzing the ratio of NSG mice with successfully induced diabetes also showed a roughly sigmoidal curve (**b**).

Figure 5a RELSA calculations to measure the severity of diabetes induction by STZ. (**a**) Time-independent blood glucose concentration in STZ-treated and control mice. (**b**) The ratio of diabetic animals for each STZ concentration. The dotted threshold line marks the 90% success rate for diabetes induction (15 mmol/L threshold). (**c**) Mean RELSA_max_ for all groups. The ANOVA and subsequent post hoc tests revealed significant differences between treated and mock-injected mice: F(5,54) = 14.12, *p* < 0.0001, with *p* < 0.01 for 150, 175, 200, and 225 mg/kg STZ. (**d**) Shown is the cross-correlation coefficient of body weight and blood glucose time series. Depicted are medians (red rhombs) and error bars (IQR). Data points represent individual data of each mouse in mock-injected mice and all treatment groups.

Here, as in males, we assumed an arbitrary 90% threshold for conversion success, so that only animals that were successfully converted and subsequently became diabetic would be used in follow-up experiments. At this threshold of 15 mmol/L, we deemed the 175 and 200 mg/kg STZ doses sufficiently effective for achieving the conversion goal (Fig. 5b).

Focusing on the cross-correlation of the time series of body weight and blood glucose changes, a positive correlation was observed in female NSG mice at low STZ doses. In contrast, the strongest negative cross-correlation between time series was achieved at the highest tested dose of 225 mg/kg, followed by 200 and 175 mg/kg STZ. Moreover, the precision of the correlation increased notably with increased dosages. At the dose of 150 mg/kg, the time-series correlation was inconclusive, as some animals gained weight despite elevated blood glucose levels. (Fig. 5d). On average, the first complete negative correlation was observed at a dose of 175 mg/kg, followed by 200 mg/kg, which also did not show any outliers.

**Fig. 6:**
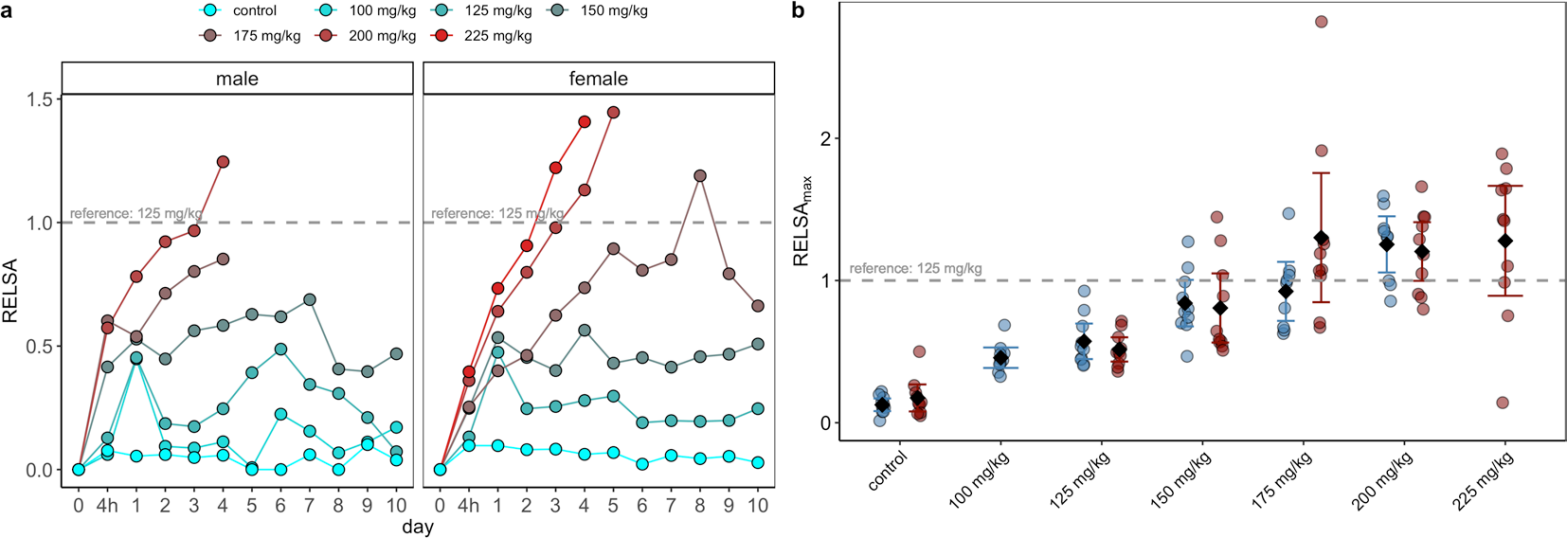
RELSA analysis of male and female NSG mice after STZ treatment. (**a**) The RELSA trajectories of females appear steeper at higher doses than those of males (data replotted from a previous study^21^ for easier comparison). In both sexes, the same RELSA reference (125 mg/kg) was used to maintain model comparability. On average, females exhibit higher severity at higher doses, such as 175, 200, and 225 mg/kg, than males. (**b**) However, no dose-dependent differences between sexes (interaction) were observed in the time-independent RELSA_max_ analysis [F_(4,106)_ = 1.67, *p* = 0.163] using a regular ANOVA. While there are some outliers among females, the 95% confidence intervals (error bars) indicate no evidence for a structural difference in maximum severity between sexes.

Before the RELSA analyses were conducted, a likelihood-ratio test compared the complete RELSA model (dose, day, and sex) against the Null model, which contained only the IDs as random effects. The IDs explained 48.42% of the total variance information (${\chi}^{2}$= 648.54, df = 57, *p* ≤ 0.0001) in the data. Therefore, we included the IDs in the subsequent dose and time-dependent analyses to account for individual differences. RELSA trajectories of female NSG mice are shown in Fig. 6a.

The study of RELSA trajectories revealed significant differences for dose [F_(5, 58)_ = 32.47, *p* < 0.0001], day [F_(10, 380.83)_ = 68.09, *p* < 0.0001; Supplementary Table 2], and the interaction between dose and day [F_(39, 379.82)_ = 11.94, *p* < 0.0001]. Each increase in STZ doses was associated with a significant increase in the RELSA value over time (Fig. 6a).

The doses of 200 and 225 mg/kg showed high average RELSA values, with the last data points exceeding the RELSA reference line of 125 mg/kg within the first 3 days of exposure, indicating a higher severity than the other doses and the maximum achieved severity at 200 mg/kg. However, the 175 mg/kg dose also exceeded the reference line on day 8, indicating a slower, yet more severe development than any lower STZ dose in female mice. In the RELSA_max_ data (Fig. 6b) of the females, a significant between-dose effect was found [F_(5, 54)_ = 14.12, *p* < 0.0001; Supplementary Table 3]. The Tukey-adjusted post hoc analyses showed significant results when the control was compared vs. 150, 175, 200, and 225 mg/kg STZ (each *p* ≤ 0.001). Further differences between doses were observed in the 125 vs. 175, 200, and 225 mg/kg groups (each *p* < 0.001).

### Dose and time-dependent severity assessment of female and male NSG mice after STZ treatment

For the comparative severity assessment of female and male mice after STZ treatment, the RELSA algorithm was used in two ways. First, the individual RELSA trajectories of male and female NSG mice were used to determine the comparative level of suffering based on two measured objective parameters: changes in blood glucose and body weight. This analysis was also conducted for the two variables, day and dose, and their interaction. Second, the average RELSA trajectories of female NSG mice were compared with those of males from a prior publication^21^ to estimate differences in the rate of severity change between sexes, also using a mixed model. The RELSA trajectories and RELSA_max_ values of males and females are shown in the facets of Fig. 6a and b. The type-III ANOVA using Satterthwaite’s method on the RELSA trajectory data in a mixed model, showed significant effects for dose [F_(6, 113.40)_ = 56.10, *p* < 0.0001], day [F_(11, 830.96)_ = 119.67, *p* < 0.0001], and sex [F_(1, 115.21)_ = 5.96, *p* = 0.02]. However, a difference between sex and dose could not be observed [F_(4, 108.76)sex: dose_ = 1.21, *p* = 0.31]. Further, the three-way interaction of dose, sex, and day was also not significant [F_(32, 828.19)dose: sex: day_ = 0.90, *p* = 0.632], indicating no average differences in dose response between sexes across time (Supplementary Table 4).

While the time-free analysis of RELSA_max_ differences in a standard ANOVA revealed no differences in the dose-sex interaction [F_(4,106) dose−sex_ = 1.7, *p* = 0.1629; Fig. 6b], the time-dependent rate-of-change differences between dose and sex interactions remained complex.

Therefore, we used a linear mixed-effects model to estimate the per-day slope *s* = Δ_RELSA_/Δ_days_ within each dose-by-sex analytical cell (Supplementary Fig. 2). In controls, slopes were *s* ≈ 0 in both sexes (female *s* = − 0.003, CI_95%_[− 0.014; 0.007]; male *s* = 0.001, CI_95%_[− 0.010; 0.013]). At 125 mg/kg, only males showed an increase (male *s* = 0.020, CI_95%_[0.008; 0.032]), whereas females did not (female *s* = 0.002, CI_95%_[− 0.008; 0.013]). At 150 and 175 mg/kg, slopes were positive in both sexes but steeper in males (150: male *s* = 0.049, CI_95%_[0.034; 0.064] vs. female *s* = 0.032 CI_95%_[0.022; 0.05]; 175: male *s* = 0.145, CI_95%_[0.110; 0.180] vs. female *s* = 0.096, CI_95%_[0.084; 0.110]). At 200 mg/kg STZ, both sexes showed large increases, with females exhibiting a slightly steeper response (female *s* = 0.240, CI_95%_ [0.210; 0.271] vs. male *s* = 0.230, CI_95%_ [0.192; 0.262]). At 225 mg/kg STZ, the slope was estimable only in females and was the largest observed (female *s* = 0.313, CI_95%_ [0.270; 0.351]). Overall, the per-day slope *s* increased with dose in both sexes. In males, the slopes were steeper up to 175 mg/kg STZ, whereas females matched or exceeded males at higher doses.

### Females require higher doses to become diabetic

To investigate the question, which optimal threshold for hyperglycemia should be used to diagnose diabetes in male and female mice, we computed the centroids for diabetic and non-diabetic groups within each dose. Further, we calculated the (Euclidean) distances between the centroids. Thresholds of 13, 14, 15, and 20 mmol/L blood glucose were evaluated separately by sex to determine the onset of diabetes. The best threshold was defined as the one that maximized balanced accuracy (BAccuracy) on a 70% training and 30% testing split, with sensitivity used to break ties. In males, the optimal threshold was at 14 mmol/L blood glucose, matching the prior male-only study and yielding the highest balanced accuracy of 98.2% (AUC = 0.999). In females, the performance at 14 mmol/L was lower than in males (91.7%, AUC = 0.976), due to reduced sensitivity at slightly lower specificity, resulting in a higher number of actual diabetic cases being missed at the same nominal dose. Focusing on dose-dependent onset, females appeared to be more resistant than males and would require higher STZ doses to achieve comparable diabetes rates. Global class separation was more minor in females than in males (13.2 vs. 17.1), quantified as the unitless Euclidean distance between diabetic and non-diabetic global centroids in the body weight change and glucose plane (Supplementary Fig. 2). Therefore, within each dose facet, the diabetic and non-diabetic centroids were more widely spaced in males, indicating a stronger dose response at the same nominal dose. In practical terms, at a fixed blood glucose threshold of 14 mmol/L, the classifier will miss more true diabetic females than males at the same dose, indicating a need for higher doses in females to achieve similar sensitivity while maintaining high specificity. These patterns were stable across thresholds in this dataset. Summary metrics are shown in Table 1.

**Table 1:** Threshold optimization for sex-specific diabetes classification.

Threshold	sex	Accuracy	BAccuracy	Sensitivity	Specificity	AUC
**20**	male	0.932	0.864	0.750	0.978	0.991
**20**	female	0.957	0.839	0.700	0.977	0.973
**15**	male	0.941	0.912	0.857	0.967	0.992
**15**	female	0.908	0.862	0.774	0.949	0.968
**14**	male	0.984	0.982	0.976	0.988	0.999
**14**	female	0.932	0.917	0.889	0.945	0.976
**13**	male	0.949	0.949	0.949	0.949	0.981
**13**	female	0.936	0.924	0.897	0.950	0.980

## Discussion

Diabetes mellitus (DM) is a metabolic disorder characterized by its primary symptom, hyperglycemia, resulting from either absolute or relative insulin deficiency and/or a defective cellular response to insulin in peripheral tissues. DM comprises heterogeneous subtypes with multiple underlying disease mechanisms. However, the two main types are type 1 and type 2 DM, which account for ~ 9% and 90% of all diabetes cases, respectively^23^.

Laboratory animals, in particular mice, play a unique and vital role in experimental DM research. The two main animal models used are spontaneous or chemically induced models of diabetes, such as the STZ model described in this study^3^. With the incidence of type 1 and type 2 DM increasing worldwide^24^, the relevance of animal models for research is also growing. Both biological sexes are equally affected by the manifestation of type 1 DM, while men are more frequently affected by type 2 DM^23^. However, distinct differences arise between women and men in the development of late complications, which can have a significant impact on life expectancy^9,10^^–^^11^^,^^14^^,^^15^.

We recently investigated the dose-dependent effect of STZ on male NSG mice, focusing on finding a dose that reliably induces diabetes while minimizing weight loss to cause as little suffering as possible^21^. The loss of body weight due to insulin deficiency places a tremendous strain on the animals. Since animal experiments are subject to moral and legal restrictions, the welfare of the animals must be strictly monitored. While moderate weight loss can be tolerated, a loss of more than 20% of body weight is considered a threshold value requiring immediate termination of the experiment^25–]^^29^. While this study pursued essentially the same objectives as before^21^, a more elaborate analysis was conducted to identify sex-specific differences in diabetes development and severity.

Female mice are known to be less vulnerable to STZ compared to males across multiple strains^6^^,^^7^^,^^30^. In previous studies, the female sex hormone estrogen and the corresponding signaling pathway were found to explain the lower sensitivity to STZ^8^^,^^31^. Therefore, as a precautionary measure, we analyzed the animals for cycle status before injecting them with STZ. Mice that were found to be in the pro-estrus cycle, when serum estrogen levels are at their peak, were injected 24–48 h later. However, we cannot judge to what extent this measure contributed to the presented results.

Of the five concentrations tested, 175, 200, and 225 mg/kg STZ produced reproducible hyperglycemia. In contrast, insufficient results were observed at 125 mg/kg STZ and 150 mg/kg STZ when using 15 mmol/L as the threshold. 175 mg/kg STZ was comparatively more effective, and the induced DM showed a slowly progressive course with manifestation between day 3 and 5 of the study. The loss of body weight was significant but remained above the 80% threshold for the most part. Only one animal had to be euthanized prematurely on day 8 of the study because of heavy weight loss. Doses of 200 and 225 mg/kg STZ caused a fulminant diabetes, passing the threshold value for blood glucose, typically already 24 to 48 h after STZ injections. The loss of body weight was equally dramatic, and judging from the kinetics of the body weight loss and the RELSA curves, would soon require euthanasia in these groups. Postmortem analysis also revealed organ damage to the kidneys and liver at 200 and 225 mg/kg STZ in several cases (data not shown). Consequently, the RELSA curves for these two concentrations were very steep, confirming the dramatic diabetes manifestation and high burden, while being only minimally more effective in causing diabetes compared to 175 mg/kg STZ. Time-resolved mean RELSA curves confirmed an increase in animal suffering over time, especially at high STZ concentrations.

The dose-response curve we selected showed a linear relationship between the mean blood glucose concentration and the STZ dose between 125 and 200 mg/kg STZ. In our study, variability in disease onset was observed primarily at intermediate doses, whereas higher doses resulted in a more rapid and consistent induction of hyperglycaemia. Importantly, higher STZ concentrations can cause hypoglycemia-induced mortality immediately after STZ injection and increased mortality in long-term experiments^3^^[,32^. Two animals died in the 225 mg/kg STZ group, one due to hypoglycemia 24 h post-injection, and another one was found dead on day 4 despite our control regimen. Further supportive care may be necessary to avoid premature animal mortality. Key measures could include an increased number of inspections including glucose and body weight measurements. Post STZ injection mortality by hypoglycemia could be reduced by intraperitoneal glucose injections. Nutritional support should be offered through energy-enriched or softened diets, with food placed on the cage floor to facilitate access for weakened animals. Housing conditions may be adapted by providing additional nesting material and maintaining appropriate ambient temperature to reduce metabolic stress. Depending on the experimental objectives, carefully controlled low-dose insulin supplementation may be used to prevent severe hyperglycemia or ketoacidosis without fully normalizing blood glucose levels. Delayed or inconsistent diabetes onset, however, necessitates prolonged and intensified monitoring, as animals may remain in a pre-diabetic or unstable metabolic state for extended periods. With respect to humane endpoints, variability in onset complicates the definition of time-based criteria and increases reliance on repeated physiological measurements.

A comparison with other studies using female NSG-mice is difficult, as the sex of the animals is often not specified^33^ or multiple low-dose STZ injections were used instead of a single high dose, as in this approach. To our knowledge, a dose-response curve for STZ has never been established in female NSG mice. However, compared to other strains, our findings are well within the typical range of the required dose. For female C57BL/6 mice, 150 mg/kg STZ was reportedly required^34^, for CD-1 mice 200 mg/kg STZ^35^, but for BALB/c mice the dose was 275 mg/kg STZ^36^.

STZ-induced diabetes models are profoundly affected by the genetic background of the mice. STZ exerts its diabetogenic effect through DNA alkylation and secondary DNA double-strand breaks^37^. The NSG mouse carries the severe combined immune deficiency genetic background Prkdc^(scid)^ with double mutations in the *Prkdc* gene, encoding DNA-dependent protein kinase catalytic subunit (DNA-PKcs). This results in a severe defect in non-homologous end joining DNA repair. Consequently, cells that had taken up STZ may exhibit markedly increased susceptibility to genotoxic stress. As a result, STZ administration in NSG mice may lead to quicker and more fulminant beta cell ablation at doses that would cause only partial damage in other mouse strains without this genetic background. Furthermore, the strong reduction of innate immune response, mostly macrophages, may further limit compensatory or regeneration-promoting mechanisms by the longer remaining cell detritus in the islets. It follows that an effective dose of STZ in another popular immunodeficient model, the NRG mouse (NOD.Cg-Rag1^tm1Mom^ Il2rg^tm1Wjl^/SzJ), would presumably be higher. Compared to male NSG mice, a slightly lower sensitivity to STZ was quickly apparent, resulting in a shifted dose-response curve in this study (125–225 mg/kg STZ) compared to males (100–200 mg/kg STZ). In terms of the effectiveness of diabetes induction, males and females showed remarkably similar results. However, to achieve a reliable diabetes induction in > 90% of the animals, 175 mg of STZ was required for females, which was 25 mg more STZ than for male animals^21^. Higher doses of STZ would lead to nearly identical efficiencies, whereby the stress on the animals would then be higher than necessary. The degranulation of insulin immunoreactivity and the different destruction processes of beta cells and the following intra-islet remodeling were comparable between islets from STZ treated pancreases from females and males with the exception of the higher doses needed in females (Fig. 4b).

We also investigated the effect of starting weight, dose, and sex (27.6 ± 2.1 g for males, 23.0 ± 1.3 g for females; SD; *n* = 56 and *n* = 60, respectively) on diabetes manifestation and found no correlation using a mixed model to account for different intercepts. The starting weight was thus irrelevant even within the sexes.

In terms of animal burden, the differences were also minimal. The increase in stress (slope of the RELSA) was higher in males at medium concentrations and greater in females at high concentrations. Overall, male mice responded with a greater increase in blood glucose levels per unit STZ than female mice. If the diagnosis of a putative diabetes was solely based on blood glucose concentration, lower doses were required for males.

In both studies, we set the threshold value for diagnosing DM at 15 mmol/L of random plasma glucose. Random plasma glucose was 6.6 ± 0.8 mmol/l (SD, 82 measurements) for healthy females and 7.6 ± 1.4 (SD, 62 measurements) for males in each control group. Fasting blood glucose values were also lower in females (Supplementary Fig. 1). The threshold value of 15 mmol/l was, therefore, approximately double the random plasma glucose concentration of the control group.

In humans, random plasma glucose testing is less clinically significant, as consuming meals with a high carbohydrate load before blood sampling can lead to artificially high values^38^. Therefore, blood samples are typically taken after an overnight fasting period. However, the normal value for a non-diabetic is ≤ 7.8 mmol/L of random plasma glucose, and the fasting blood glucose value is considered normal when it is lower than 5.6 mmol/L^38^. However, the threshold value for a DM diagnosis is significantly lower at only 11.1 mmol/L random plasma glucose^38^. Interestingly, based on the data we collected, we were able to demonstrate that a threshold of 14 mmol/L is most likely to distinguish healthy male animals from those with diabetes. In contrast, only 13 mmol/L is required for female mice.

We used a generalized linear model to construct a classifier with randomly sampled training data. We then used the remaining data to prevent information leakage and to test the classifier with body weight change and blood glucose as predictors. Interestingly, we not only demonstrated that the Euclidean distances between body weight change and glucose centroids were consistently smaller in females than in males, but also found that this led to more false-negative results at lower doses and more false-positive results at higher doses at a given threshold, e.g., 14 mmol/L. Adjusting the threshold improved the classification result, indicating that at the same threshold, females consistently require higher doses due to a lack of sensitivity (Table 1). In other words, we can demonstrate that using both measures, body weight change and blood glucose, can equally well separate negative diabetes findings; however, it performs worse in identifying positives for females. However, while these results support previously reported findings, generalizations based on this machine learning approach beyond this experiment should be cautious, given the limited dose range and within-study train-test splits, as well as the limited sample size.

This study has limitations that should be considered when interpreting the dose–response effects of STZ in mice. First, the STZ concentrations described here may not be transferable to other mouse strains due to the specific genetic background of the NSG mouse. Consequently, the findings may not be directly usable in immunocompetent or Rag-deficient models. Second, the short observation period of ten days captured only acute effects of STZ exposure and precludes assessment of delayed toxicity and metabolic stabilization by potential beta cell recovery. Long-term consequences on body weight, glycemic control, and animal welfare therefore remain unknown.

## Conclusion

In summary, we can show that a single dose of 175 mg/kg STZ per kg body weight can reliably induce DM in female NSG mice. Higher concentrations were not profoundly more effective but produced unnecessary animal suffering, as shown by the RELSA data using the two input parameters, blood glucose concentration and body weight. In direct comparison with males, females showed slightly lower sensitivity to STZ, although the difference was relatively small. The analysis using RELSA revealed a comparable severity of the animals when comparing dose and sex. We therefore conclude that female STZ-treated animals are equally suitable in terms of effectiveness and reproducibility for use as a model of diabetes.

## Materials and methods

### Housing

Housing and experimentation of mice in this study were conducted in accordance with the regulations of the German Animal Welfare Act and the ARRIVE guidelines^39^^[,40^. The experimental procedures were performed as previously described by Talbot and colleagues^21^ with minor modifications.

In detail, female NOD.Cg-Prkdc^scid^ Il2rg^tm1Wjl^/SzJ (NSG) mice aged 8–12 weeks from in-house breeding (Jackson laboratory, strain #005557) of the Institute for Laboratory Animal Science of the Hannover Medical School were housed within ventilated cabinets (Scanbur, Karlslunde, Denmark) in individual cages equipped with filter bonnets. The cages were furnished with autoclaved softwood granulate (poplar wood, AB 368P, AsBe-wood GmbH, Buxtehude, Germany) as nesting material, along with autoclaved cotton rolls (ANT Tierhaltungsbedarf, Buxtehude, Germany) and enrichment materials (igloo, gnawing material). Housing and experimentation were performed in a specific pathogen-free room at 21 °C and 50% humidity, following a 12:12-h light/dark cycle. The mice had unrestricted access to drinking water and a gamma-irradiated (25 kGy) standard breeding diet (Altromin TPF-1324, Lage, Germany). While the diet was removed from the cages for 4 h to induce hunger in the animals prior to STZ injection, drinking water remained accessible. All human interactions, including handling, weighing, cage maintenance, i.p. STZ injections, blood glucose measurements, and scorings were carried out under a laminar flow utilizing sterile tools to ensure aseptic conditions.

### STZ injection and diabetes induction

Experimental groups were allocated randomly by technical staff who were not involved in the planning and experimental steps of the study. The animals’ baseline starting weight at the beginning of the experiment was 23 ± 1.3 g (SD, *n* = 60). Before STZ injections, the mouse estrous cycle stage was determined by collecting vaginal cells^41^. Briefly, the vaginal canal was rinsed 3–4 times with 100 µl sterile H_2_O using a latex bulb on a sterile 200 µl tip. The collected fluid was then placed on a glass slide, heat-dried, and fixed with 96% ethanol. The fixed smear was then stained with methylene blue for 2 min, followed by two washing steps with H_2_O. After mounting, the slide was examined under light microscopy to determine the cell types present.

STZ injections were prepared by dissolving STZ (Santa Cruz Biotechnology, U-9889, Dallas, Texas, USA; α-anomer content, 88.78%) in concentrations ranging from 12.5 to 22.5 mg/mL in sodium citrate-acidified PBS, pH 4.5. The freshly prepared solution was then immediately administered within five minutes always at 11 o’clock. a.m for all groups. STZ was injected intraperitoneally (i.p.) in female mice outside the proestrus cycle that had fasted for 4 h from 7 to 11 o’clock in the morning. The dosage ranged from 125 to 225 mg of STZ per kg body weight. Body weights were measured the day before the experiment and immediately before the STZ injection to ensure precise calculation of the required STZ dose using a baseline value.

Blood glucose concentrations were monitored before (fasting blood glucose) and 4 h after STZ injection, using tail-tip blood samples measured in a Contour Next glucometer (Ascensia Diabetes Care, Basel, Switzerland). To reduce hypoglycemia-induced mortality, the mice were offered a sterile-filtered 10% sucrose solution for 24 h instead of their standard drinking water. Diabetes was diagnosed when blood glucose levels (random blood glucose) exceeded 15 mmol/L in consecutive measurements on different days. The study design is depicted in Fig. 1 and spanned over 10 days.

### Immunohistochemistry

Pancreases were fixed in 4% paraformaldehyde, embedded in paraffin and sectioned in 2 μm slices. After removal of the paraffin, staining was performed overnight with a primary antibody against insulin (Agilent Dako, Santa Clara, USA, polyclonal guinea pig A564 dilution 1:400,). Biotinylated goat anti-guinea pig Ig G (1:400; 30 min) and a streptavidin-biotin-peroxidase complex (1:1,000; 30 min) (both from Jackson Immuno Research, West Grove, IL, U.S.A.) were used as a secondary antibody. The peroxidase was visualized with 0.7 mM diaminobenzidine and 0.002% H_2_O_2_ in 0.05 mM Tris HCl buffer, pH 7.6. Images were taken on an Olympus BX61 microscope.

### Animal scoring

The animals underwent bi-daily scorings, which included daily assessments of both body weight and blood glucose levels following the manifestation of diabetes. First assessment was performed at 11 o’clock a.m. followed by a second assessment in the afternoon.

A specific table for the body scoring system was used, which comprised the general appearance, facial expression, body posture, activity, behavior, and polyuria/polydipsia (Supplementary Table 1). Non-diabetic animals were scored at least three times a week, with concurrent measurements of blood glucose and body weight. In case of rapid and severe diabetes progression, particularly at higher STZ concentrations, animals were euthanized no later than 96 h after STZ injection or 96 h after diabetes manifestation. All diabetic animals were offered softened chow from the day of diagnosis, which was provided in a Petri dish on the floor of the cage.

To ensure animal welfare and compliance with German regulatory requirements, two consecutive blood glucose measurements exceeding 30 mmol/L were considered as a criterion for study termination, owing to the potential risks associated with life-threatening hypovolemia and/or ketoacidosis. A second criterion for immediate discontinuation was a loss of over 20% of initial body weight in accordance with German authorities’ and international guidelines^27^^,^^28^. Euthanasia was conducted no later than 10 days after the initiation of the experimental protocol by STZ injection. For this, the mice were anaesthetized with CO_2_ in their home cages, which had an area of 530 cm^2^ and a volume of approximately nine and a half litres. Using a flow meter, the cages were gradually filled at a flow rate corresponding to 20–25% of the cage volume per minute, in this case 2 L/min. After complete cessation of breathing, the mice were bled by cardiac puncture and death was additionally ensured by cervical dislocation. The pancreases were then extracted for subsequent histological analyses. The differences in the conduct of this study compared to the comparator male group were as follows: (1) the previous study^21^ was conducted on male animals without addressing the influence of male sex hormones or the lack of estrogen, and (2) the dose-response curve was shifted by 25 mg from 125 to 225 mg STZ per kg BW in female mice compared to 100–200 per kg BW in male mice. All other methods were identical and were performed by the same personnel and in the same premises.

### Statistics and RELSA analysis

The following statistical and RELSA analysis was carried out using our methods described by Talbot and colleagues^21^ with minor modifications.

Statistical analyses of individual parameters presented in Figs. 2 and 3, and 4 were carried out using the GraphPad Prism analysis software (GraphPad, San Diego, CA, USA). The unpaired *Student’s t*-test was employed, and the results were corrected for multiple comparisons using the Bonferroni-Dunn method, resulting in adjusted p-values denoted as *** for *p* ≤ 0.001, ** for *p* ≤ 0.01, and * for *p* ≤ 0.05. The log-rank (Mantel-Cox) test was used to compare the rates of diabetes manifestation with those of the control. RELSA results were analyzed using a linear mixed-effects regression (lmer) to estimate the time- and dose-dependent effects and their interaction, utilizing the lme4, lmerTest, emtrends, and emmeans R packages. The animal ID was integrated as a random effect to account for the within-subjects correlation. It is important to note that the analysis excluded data from the 4-hour time point on day 0. The significance of the animal ID was assessed using a likelihood-ratio test against the Null model. From this model, we estimated per-day slopes *s* = Δ_RELSA_/Δ_day_ for each dose-by-sex stratum. For each stratum, we tested *s =* 0 and reported point estimates with 95% CI intervals. Slope differences between sexes within dose and between doses within sex were assessed via pairwise contrasts of trends with Holm’s adjustment for multiplicity. Strata with insufficient information were labeled “nonEst.” Finally, the coefficients were subsequently transformed into an ANOVA table, enhancing their readability by utilizing Satterthwaite’s method to approximate the degrees of freedom in the presence of interactions.

The RELSA procedure^22^ was used to fuse the two-dimensional, normalized input data of glucose and body weight change into a single scalar for relative severity comparisons. This analysis enabled the relative comparison of the analyzed animals with a reference. In any case, the reference group was mice at the 125 mg/kg dose. So, when animals showed values above RELSA = 1, this corresponded to larger escalations in the input variables than in the reference set. The individual RELSA values were used to generate averaged and time-dependent RELSA trajectories. Additionally, a RELSA_max_ analysis was employed to identify time-independent maximum severity values in each animal, facilitating the identification of group-specific severity or extreme values.

For the analysis of time-independent RELSA_max_ results, a linear model was employed. Post hoc tests were used to estimate between-dose contrasts, with adjustments made using the Tukey procedure to mitigate family-wise errors. Results were reported as an ANOVA table and post hoc contrasts. Furthermore, we examined the zero-lag cross-correlation of the individual time series of body weight change and blood glucose levels for each STZ dose administered. The specific coefficients were averaged and plotted to investigate the average parameter dominance at each STZ dose.

Finally, we combined male and female datasets, retained IDs, dose, sex, day, body weight change, glucose, and a diabetic flag defined from glucose thresholds of 13, 14, 15, and 20 mM using a confirmation rule that required the current and at least one prior value to meet the threshold, and excluded measurements taken at day 0 at 4 h. For visualization, we plotted glucose against body weight change by dose and sex, computed the centroids for diabetic and non-diabetic groups within each dose, calculated the Euclidean distances between the centroids, and added sex-specific linear decision boundaries from a logistic regression model with glucose and body weight change as predictors. We also computed the global centroid distances by sex across doses. Candidate thresholds were compared using random 70% training and 30% test splits with a fixed seed. The performance of each model was evaluated separately by sex using the same split and reported as accuracy, balanced accuracy, sensitivity, and specificity.

## Supplementary Information

Below is the link to the electronic supplementary material.

Supplementary Material 1

## Data Availability

Raw data used and/or analyzed in this study can be obtained from the corresponding author upon request.
