# Supplementary material for: Comparative analysis of dose-response variability and severity in STZ-induced diabetes: female vs. male NSG mice
Source: Sci Rep. 2026 Mar 5;16:8257. doi: 10.1038/s41598-026-42408-z (PMC12963432; doi:10.1038/s41598-026-42408-z)
Supplement: Supplementary file 1 — Supplementary Material 1 [file 41598_2026_42408_MOESM1_ESM.docx]

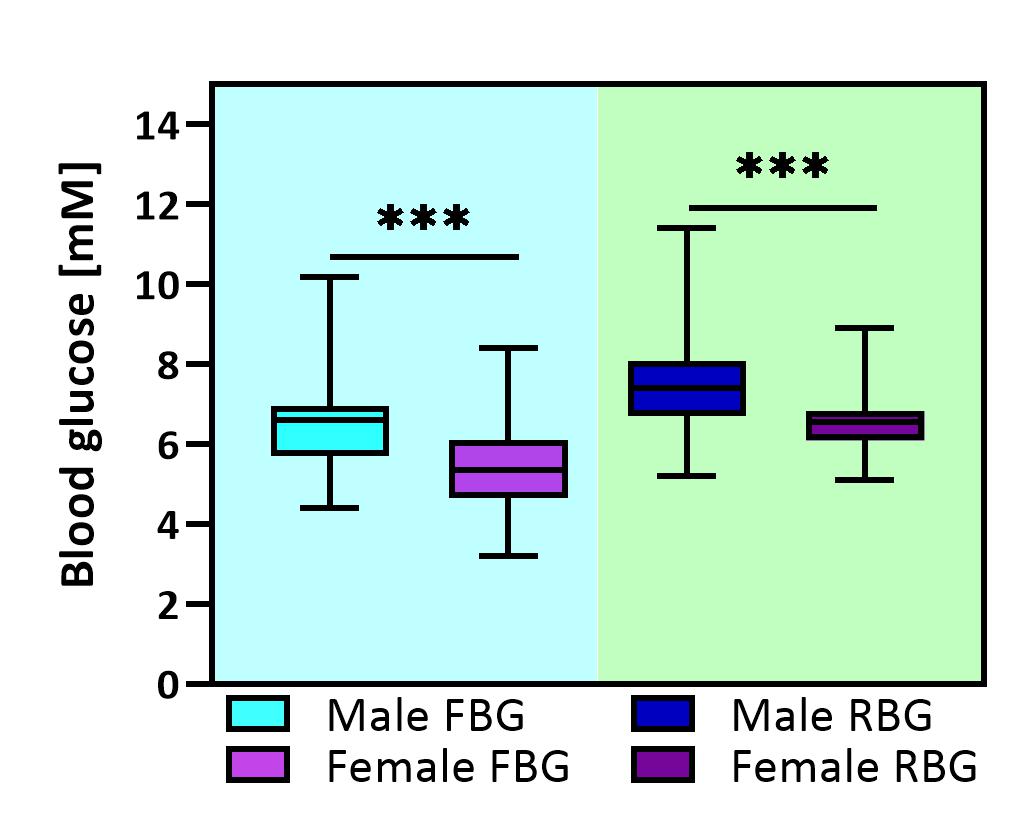


**Supplementary Figure 1: Comparison of fasting and random blood glucose in male and female NSG-mice.**

Blood glucose was measured by tail-tip puncture and determined using a Contour Next glucometer. Male data from Talbot et al. Exploring dose–response variability and relative severity assessment in STZ-induced diabetes male NSG mice. Sci Rep 14, 16559 (2024). Fasting blood glucose (FBG) was measured after a 4-hour fasting period in all animals. Random blood glucose (RBG) levels were measured in 10 control animals over a 10-day period. Data are presented as a Box-Whisker plot (min-max), n=60-81. Unpaired two-tailed *Student’s* t-test, *** = p ≤ 0.001.


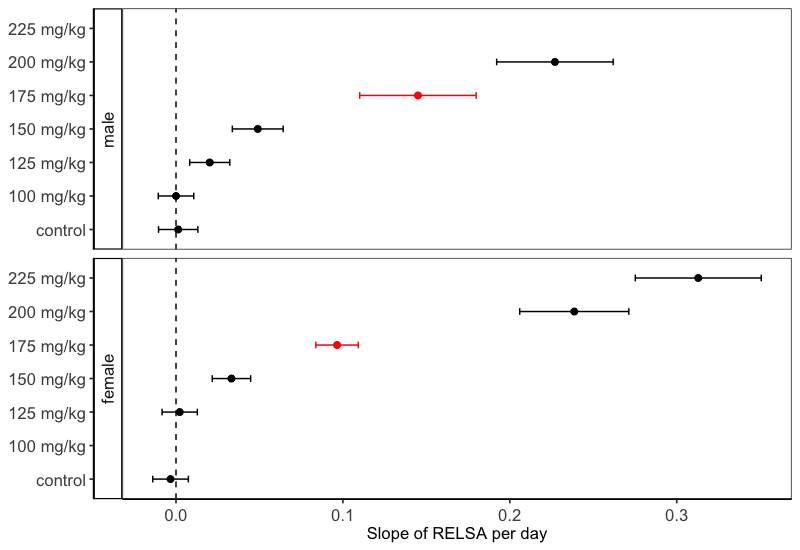


**Supplementary Figure 2: Differences in the rate of change in severity by dose and sex.**

Each point represents the estimated daily change in RELSA. Horizontal bars are 95% confidence intervals, and the dashed vertical line marks “no change.” Panels are divided by sex, and the y-axis lists the doses. The 175 mg/kg dose (highlighted in red) shows clear increases in both sexes, with different slopes: approximately 0.096 per day in females (CI_95%_ [0.084; 0.109]) and 0.145 per day in males (CI_95%_ [0.110; 0.180]). The steeper increases generally occur at higher doses. Estimates for 100 mg/kg in females and 225 mg/kg in males were not estimable and are omitted. Estimates come from a mixed-effects model with animal as a random effect.

**
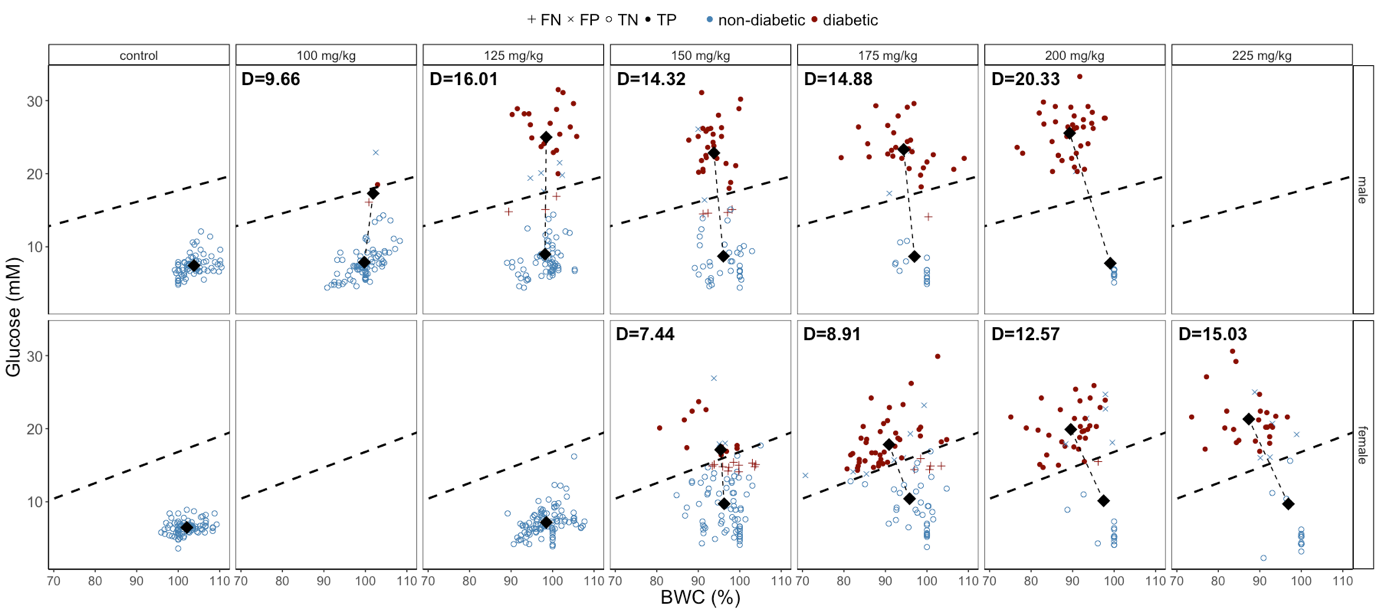
**

**Supplementary Figure 3: Classification of diabetes based on a fixed threshold of 14 mM glucose using a linear classifier.**

Scatterplots by dose and sex show body weight change (%) vs. glucose. Color = observed status (non-diabetic/diabetic), shape = model outcome (TP/TN/FP/FN). The dashed black line represents the sex-specific logistic 50% decision boundary, determined by a model based on a randomly selected training set. Black “◆” marks class centroids, and the dashed connector with D gives their Euclidean distance (larger = more apparent separation). Performance for alternative thresholds is reported in Table 2, which is provided in the accompanying document.

**Supplementary Table 1: Body scoring system used in the study.**

| **Score** | **Activity** | **Body weight** | **General condition** | **Behavior** | **Measures** |
| --- | --- | --- | --- | --- | --- |
| **1** | Very active | +/- 5 % | Fur smooth and shiny; eyes clear and shiny;  Body orifices clean | Lively, attentive, curious, movements typical of the species | Checks:  Day -1 to day 4: daily from day 5 on: 3x per week |
| **2** | Active | 5-<10 % | Fur smooth and shiny; eyes clear and shiny;  Body orifices clean | Lively movements typical of the species, signs of PU/PD, polydipsia  Blood glucose  > 7 and < 15 mmol/l | Daily checks |
| **3** | Calm, less active | 10-<20 % | Fur smooth and shiny; eyes clear but almond-shaped, shiny;  Body orifices clean | Alert, somewhat calmer, reduced movement, posture typical of the species, evident signs of PU/PD, blood glucose  >15 mmol/l | Twice daily checks, soaked food on the cage floor  If necessary, additional measurement of blood glucose  The experiment was stopped after 96 hours in diabetic animals  Immediate termination if BGW >30 mml/l in two consecutive measurements |
| **4** | Limited activity | ≥ 20 % | Fur dull, erect; eyes no longer fully open; body orifices untended | Animal calm, frequent lingering, slightly curved back line, reduced personal hygiene, limited reactions to environmental stimuli, and blood glucose  >15 mmol/l | Immediate termination of the experiment as soon as one criterion from score 4 is recorded |
| **5** | No activity, lethargic | ≥ 20 % | Fur erect, dirty; eyes closed; body orifices dirty or moist | Self-isolation; strongly curved back line, no significant activity, hardly any reaction to environmental stimuli  blood glucose >30 mmol/l | Immediate termination |

PU = polyuria, PD = polydipsia

**Supplementary Table 2: Type III ANOVA Table of female RELSA trajectories.**

|  | **SS** | **MSS** | **NumDF** | **DenDF** | **F-value** | **Pr(>F)** | **stars** |
| --- | --- | --- | --- | --- | --- | --- | --- |
| **Dose** | 4.73 | 0.95 | 5 | 58 | 32.467 | 1.25*10^-15^ | *** |
| **Day** | 19.86 | 1.99 | 10 | 380.83 | 68.085 | < 2.2*10^-16^ | *** |
| **Dose:Day** | 13.58 | 0.35 | 39 | 379.82 | 11.937 | < 2.2*10^-16^ | *** |

**Supplementary Table 3: ANOVA Table of female RELSA_max_.**

|  | **DF** | **SS** | **MSS** | **F-value** | **Pr(>F)** | **stars** |
| --- | --- | --- | --- | --- | --- | --- |
| **Dose** | 5 | 11.18 | 2.24 | 14.12 | 7.88*10^-9^ | ******* |
| **Residuals** | 54 | 8.55 | 0.16 |  |  |  |

**Supplementary Table 4: ANOVA table of between sex comparisons of RELSA-related, comparative severity assessments of NSG mice after STZ treatment.**

|  | **SS** | **MSS** | **NumDF** | **DenDF** | **F-value** | **p-value** | **stars** |
| --- | --- | --- | --- | --- | --- | --- | --- |
| **dose** | 7.94 | 1.32 | 6.00 | 113.40 | 55.10 | < 0.0001 | *** |
| **sex** | 0.14 | 0.14 | 1.00 | 115.21 | 5.96 | 0.02 | * |
| **day** | 31.05 | 2.82 | 11.00 | 830.96 | 119.70 | < 0.0001 | *** |
| **dose:sex** | 0.11 | 0.03 | 4.00 | 108.76 | 1.21 | 0.31 |  |
| **dose:day** | 22.70 | 0.41 | 55.00 | 829.21 | 17.50 | < 0.0001 | *** |
| **sex:day** | 0.54 | 0.05 | 11.00 | 829.69 | 2.07 | 0.02 | * |
| **dose:sex:day** | 0.68 | 0.02 | 32.00 | 828.19 | 0.90 | 0.63 |  |
